# Stem Cell Therapy for Corneal Epithelium Regeneration following Good Manufacturing and Clinical Procedures

**DOI:** 10.1155/2015/408495

**Published:** 2015-09-16

**Authors:** Beatriz E. Ramírez, Ana Sánchez, José M. Herreras, Itziar Fernández, Javier García-Sancho, Teresa Nieto-Miguel, Margarita Calonge

**Affiliations:** ^1^Institute of Applied Ophthalmobiology (IOBA), University of Valladolid, Campus Universitario Miguel Delibes, Paseo de Belén 17, 47011 Valladolid, Spain; ^2^Institute of Molecular Biology and Genetics (IBGM), University of Valladolid, Valladolid, Spain; ^3^Networking Center for Biomedical Research in Bioengineering-Biomaterials and Nanomedicine (CIBER-BBN), Carlos III National Institute of Health, Spain

## Abstract

*Objective*. To evaluate outcomes of cultivated limbal epithelial transplantation (CLET) for management of ocular surface failure due to limbal stem cell deficiency (LSCD). *Design*. Prospective, noncomparative, interventional case series and extensive comparison with recent similar studies. *Participants*. Twenty eyes with LSCD underwent CLET (11 autologous; 9 allogeneic) and were followed up for 3 years. Etiologies were divided into 3 prognostic categories: Group 1, chemical injuries (7 eyes); Group 2, immune-based inflammation (4 eyes); and Group 3, noninflammatory diseases (9 eyes). *Intervention*. Autologous and allogeneic limbal epithelial cells were cultivated on amniotic membranes and transplanted. Evaluations were based on clinical parameters, survival analysis, and in vivo confocal microscopy (IVCM). European Union Tissues/Cells Directive and good manufacturing procedures were followed. *Main Outcome Measures*. Improved clinical parameters, absence of epithelial defects, and improved central corneal epithelial phenotype. *Results*. Success rate was 80% at 1-2 years and 75% at 3 years. Autografts and allografts had similar survival. Success rate was significantly lower in prognostic Group 1 (42.9%) than in Groups 2-3 (100% each). All clinical parameters improved substantially. By IVCM, 80% of cases improved in epithelial status. *Conclusions*. CLET improved corneal epithelium quality, with subsequent improvement in symptoms, quality of life, and vision. These results confirm that CLET is a valid therapy for ocular surface failure.

## 1. Introduction

Corneal epithelial stem cells reside throughout the entire circumference of the corneoscleral limbal niche that includes the limbal crypts of the palisades of Vogt. They are responsible for the maintenance of a healthy corneal epithelium, in part, by replacing aged or damaged epithelial cells during normal cell turnover [1–3]. A deficiency or lack of corneal epithelium renewal due to limbal epithelial stem cell depletion or dysfunction results in the so-called limbal stem cell deficiency (LSCD) syndrome, which is a difficult and complex disorder to manage when it is complete and severe. The hallmark of the LSCD phenotypic endpoint is the replacement of the corneal epithelium by conjunctival epithelium. With the loss of the limbal epithelial stem cells, the limbal conjunctival epithelial cells invade the superficial cornea. In LSCD, the unstable ocular surface causes recurrent corneal epithelial breakdown or nonhealing ulceration and vascularization associated with chronic inflammation. These surface alterations result in pain, photophobia, decreased vision, and eventual corneal blindness [1, 4–10]. This syndrome engenders a high risk for corneal graft failure as the donor graft (or artificial cornea) does not have epithelial limbal cells. Likewise, amniotic membrane transplantation, although useful in partial LSCD cases, is inadequate for total LSCD, requiring the addition of limbal tissue [11].

Logically, the definitive treatment for medically irreversible total and/or severe LSCD is the transplantation of limbal tissue or limbal epithelial cells. This approach repeatedly has been shown to help ocular surface regeneration, and consequently it improves the prognosis of a subsequent corneal graft [1, 4–10].

Other types of cells are being tested at present and some have reached the human clinic. For instance, the use of autologous conjunctival cells cultivated ex vivo on amniotic membrane has been recently published [12]. Nonocular mucosal epithelial cells such as those from the oral mucosa have provided non-stem cells resources for use in humans [13, 14], and there are ongoing clinical trials with this approach. Stem cells derived from nonocular mucosal or nonepithelial sources have not yet reached the human clinic, although there are clinical trials already registered on the potential use of mesenchymal stem cells in LSCD (https://www.ClinicalTrials.gov/ Identifier: NCT01562002). Finally, the exciting possibility of using induced pluripotent stem cells as a source of limbal epithelial stem cells with translational potential has just begun [15].

The encouragement for limbal epithelial transplantation was initiated with whole limbal transplantation, first performed in the treatment of severe unilateral ocular surface disease due to LSCD in 1989 by Kenyon and Tseng [16]. At present, whole limbal transplantation is performed through a limbal biopsy, usually of the 4–6 o'clock hour region from the healthy eye in unilateral LSCD cases [17]. In cases of bilateral LSCD, limbal transplant tissue is usually derived from a living relative or a cadaveric donor. At present, these procedures are performed mainly in the United States of America (USA). The so-called “Cincinnati procedure” combines conjunctival limbal allograft and keratolimbal allograft from a living relative of the patient or the same modified technique in which tissues are autologous [18].

A novel surgical technique for unilateral LSCD was recently published by Sangwan et al. [19]. This technique, called “simple limbal epithelial transplantation,” extracts a small autologous biopsy of whole limbal tissue from the contralateral healthy eye. The biopsy tissue is divided into 8–10 pieces, placed on top of fresh human amniotic membrane already glued by fibrin to the scraped corneal bed in the diseased eye. A modification of this technique has just been published to make this procedure available in the USA by using cryopreserved amniotic membrane (American Food and Drug Administration [FDA] approved) in a double layer that sandwiches the limbal cells [20].

An alternative to whole limbal tissue transplantation is to use stem cell therapy, where stem cells and other cells resident in the niches are isolated and expanded. For corneal epithelium reconstruction, the technique is called “cultivated limbal epithelial transplantation” (CLET), first reported successful in two cases by Pellegrini et al. in 1997 [21]. In this procedure, the donor limbal tissue is cultivated in vitro, allowing the cells to proliferate. The required amount of tissue is fairly small (1-2 × 1-2 mm limbal biopsy), which decreases the risk of LSCD in the donor eye in unilateral cases. It also makes it possible, at least in theory, to remove the small biopsy even if the contralateral donor eye has some limbal damage. When the disease is bilateral and the LSCD is complete, the transplant must be allogeneic. Because the source of the expanded cadaveric tissue can be small and the cultured limbal stem cells may have unique immunoprotective properties [3, 22], patients subjected to CLET require lower doses of immunosuppressive therapy for a shorter period of time than those undergoing whole limbal transplantation.

Currently it is not clear if autologous and allogeneic CLET have similar success rates. There are also very few reports that compare CLET with other means of transplanting limbal tissues. The only report comparing CLET with whole donor limbal transplantation suggests that CLET is superior to conventional techniques [23]. These data are based on each procedure being performed separately on each eye of the same patient. Currently there are no studies that compare CLET with simple limbal epithelial transplantation.

Since that first report in 1997 [21], the long-term restoration capacity of the ocular surface by CLET has been demonstrated in many reports, and it is more widely used at present. The CLET procedure is now approved in the European Union (EU), provided that it follows the EU Tissues and Cells Directive regarding good manufacturing procedures (GMP). Further, each transplantation medical institution must receive permission from each national regulatory agency. In the USA, there is no FDA approval for the use of GMP facilities to process and cultivate the autologous or allogeneic pieces of limbus. Consequently, there are not many reports on the long-term efficacy of CLET procedures that follow the most stringent regulations and GMP that, at least in the EU, equate cell therapy to any other drug therapy.

We report herein the long-term outcome of both autologous and allogeneic stem cell therapy by CLET. The study was performed in compliance with the EU Tissues and Cells Directive for reestablishing the corneal epithelium phenotype in cases of ocular surface failure due to total or severe LSCD caused by three groups of diseases: chemical injuries, immune-based inflammation, and other less severe diseases. As there are few studies published following these requirements we have extensively compared our results with others using similar approaches.

## 2. Methods

### 2.1. Patients

Twenty eyes of 19 Caucasian patients diagnosed with LSCD were treated by CLET (Table 1). The protocol was approved by the institutional review board of IOBA-University of Valladolid. Additional approval was obtained from the University of Valladolid Ethics Committee. Informed consent was granted by all patients after full explanation of all procedures. All experiments and procedures were conducted in accordance with the GMP and good clinical practice norms and the EU Tissues and Cells Directive. The Declaration of Helsinki and the provisions of “National Organic Law 15/1999 of 13 December on the Protection of Personal Data” (BOE number 298, 14-12-1999, pp. 43088–43099) were followed in terms of patient rights, identification, data management, and statistical analysis. As this study was not a clinical trial but rather a clinical series study, it was not registered in the clinical trial databases. However we have now registered a clinical trial that has just finished recruitment (https://www.clinicaltrials.gov/).

LSCD diagnosis was confirmed at the initial visit based on (1) clinical signs under slit lamp examination (SLE), including absence of the normal appearance of the limbal area and at least 3 of the following corneal signs: central epithelial irregularity ≥2 (range 0–3), central epithelial opacity ≥2 (range 0–4), superficial neovascularization (area and length of neovessels) ≥3 (range 0–4), and recurrent or persistent epithelial breakdown, all as defined below; (2) conjunctival-like or mixed epithelial cell phenotype in the central cornea (as defined below) observed by in vivo confocal microscopy (IVCM). All patients had already been through medical therapies intended to quiet their ocular surface and reverse as much as possible any treatable limbal dysfunction.

Grading of LSCD was made by clinical evaluation based on parameters previously published [24, 25]. We selected only patients with total LSCD, in which the cornea was completely vascularized and opacified, or severe LSCD, in which there were recurrent or persistent epithelial defects, peripheral corneal conjunctivalization involving more than 2 quadrants, and central cornea opacification. Moderate cases of LSCD with sectorial conjunctivalization involving less than 2 quadrants, none of which reached the central cornea, were not considered for this study.

The final outcome of CLET strongly depends on the etiology of LSCD, among other factors. Thus, the candidate eyes were grouped into one of the following three etiological categories at the initial visit: Group 1 had chemical injuries (only included at a minimum of 12 months after the acute trauma and after other therapies had failed); Group 2 had immune-based inflammatory diseases (i.e., cicatrizing conjunctivitis such as Stevens-Johnson syndrome, mucous membrane pemphigoid, atopic keratoconjunctivitis, rosacea-related keratoconjunctivitis); and Group 3 had other noninflammatory conditions such as sequelae from multiple surgeries, chronic sequelae of infectious keratitis already sterile, congenital aniridia, contact lens wear-related, and so forth.

Although limbal stem cells may have immunosuppressive properties [3, 22], immune rejection has been reported after allogeneic CLET [26]. Thus, to prevent any possible immune rejection, patients who were to receive allogeneic CLET were started at the initial visit on oral immunosuppressive therapy with one of the following drugs: 1.5–2.0 g/day of mycophenolate mofetil, 3–5 mg/kg/day of cyclosporine A, or 1-2 mg/kg/day of azathioprine. The immunosuppressive treatment was maintained for 12 months after CLET, after which the drug was tapered and discontinued in the next 3 months. We closely monitored potential side effects at each visit, by phone if necessary and by blood pressure and blood/urine workup every 1-2 months.

Five to 7 days after the initial visit, unilateral LSCD cases who agreed to undergo autologous CLET underwent a limbal biopsy in the contralateral healthy eye. The limbal cells were grown in culture for 3–5 weeks, and the CLET procedure was then performed using the harvested cells. Bilateral cases had allogeneic CLET from cadaveric donors between 4–6 weeks after the initial visit so that the elapsed times between that visit and CLET were similar in all patients.

Patients were then evaluated 24 hr. after CLET, weekly for the first month, monthly until the sixth month, and every other month up to the first year. After the first year, patients were evaluated clinically every 6 months for 2 more years, reporting clinical and photographic outcomes up to three years after CLET.

Prior to the initiation of these procedures, all patients were screened for the mandatory transmittable diseases: human immunodeficiency virus, human T-cell leukemia-lymphoma virus, hepatitis C and B virus, and syphilis. All allogeneic tissue donors (cadaveric limbus and human amniotic membrane) were similarly screened.

### 2.2. Cultivation and In Vitro Expansion of Limbal Epithelial Cells

Cells destined for CLET were cultured at the University of Valladolid Institute of Molecular Biology and Genetics Cell Processing Unit that was licensed and accredited by the Spanish Agency of Medicines and Sanitary Products (AEMPS). The institute operates under GMP regulations and holds a protocol registered as PEI 09-137 by the AEMPS.

**Figure 1:**
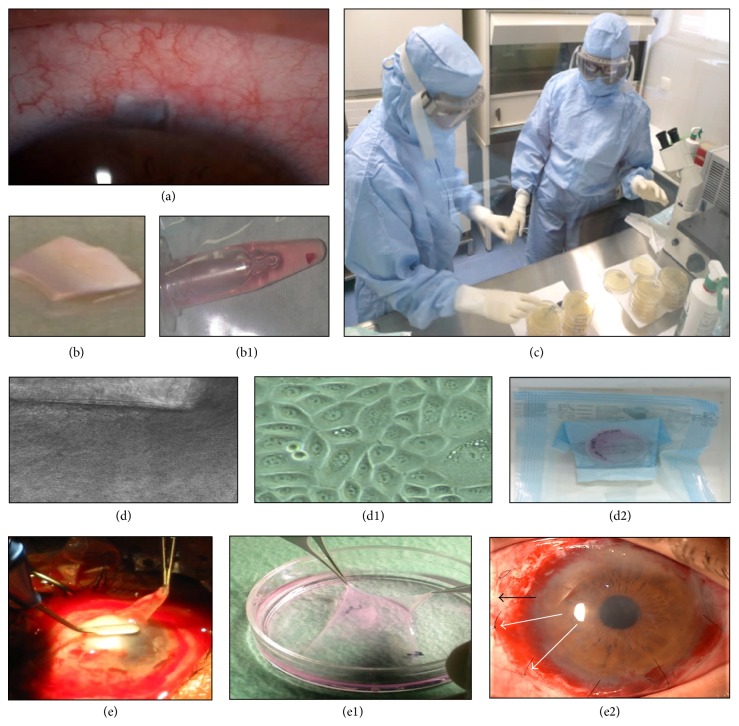
Limbal biopsy, limbal epithelial cultivation, and cultivated limbal epithelial transplantation (CLET). (a) Healthy donor eye 24 hr. after a 2 × 2 mm limbal biopsy; (b) the biopsy tissue was placed in an Eppendorf tube with culture medium (b1); (c) the biopsy was processed in a good manufacturing practice-cell processing unit within the next 4 hr. and for the next 4-5 weeks; and (d) the explant was placed on denuded human amniotic membrane, as viewed by contrast phase microscopy. Limbal epithelial cells began outgrowth from the explant at 1-2 weeks (d1). The explant was then removed, and the outgrowth was maintained until reaching confluence at which time it contained approximately 250,000 cells. The cell product was then sent to the medical center for CLET (d2). (e) Superficial keratectomy in the diseased contralateral eye (Case 1); (e1) human amniotic membrane with epithelial limbal cells confluent on top is removed from culture dish; (e2) the complex of amniotic membrane-limbal stem cells is placed on top of the previously denuded corneal and sclerolimbal surface; the amniotic membrane limit is observed (black arrow) with cells facing down and sutured (white arrows). A scleral lens is then applied.

Donor human amniotic membranes and cadaveric limbal rings came from a registered and accredited tissue bank (Blood and Tissue Community Center, Oviedo, Asturias, Spain) and were screened for transmittable diseases as described above. Allogeneic limbal rings were obtained within 7 days of death from corneal donors who were under 60 years old. Autologous limbal biopsies were marked by the surgeon with a stitch in the upper right corner to ensure consistency when plating the explant to ensure the same way up; they were sent from the operating room to the cell production unit at 4–10°C in culture medium (Figure 1).

Human amniotic membranes were prepared using our standardized protocol. After thawing the membranes previously stored at −80°C, they were washed with phosphate buffered saline (PBS) (Life Technologies-Gibco, Madrid, Spain) and treated with trypsin (Life Technologies-Gibco) for 15 min at room temperature. This was followed by gentle cell scraping to separate the epithelial cells from the underlying stroma. Once deepithelialization was complete, the amniotic membrane stroma was washed twice in PBS to remove cellular debris. It was then attached to the bottom of a 35 mm cell culture dish so that the basement membrane faced upwards to serve as a substratum for the limbal epithelial cells.

Limbal tissues were processed during the 4 hr. following arrival (Figure 1). Either the autologous limbal explant or one piece of allogeneic limbal tissue (2 × 2 mm each) was cultured on top of the denuded amniotic membrane. The culture was performed initially under a drop of fetal bovine serum (FBS) (Life Technologies-Gibco), in standard conditions of 37°C, 95% humidified air/5% CO_2_ gas mixture. After 24 hr., 3 mL of the following culture medium was added: DMEM/F12 media (1 : 1 mixture) (Life Technologies-Gibco), 5% FBS (Life Technologies-Gibco), 50 *μ*g/mL hydrocortisone (Sigma Aldrich, St Louis, MO, USA), 0.5 ng/mL cholera toxin (Gentaur, Kampenhout, Belgium), 5 ng/mL insulin-transferrin-selenium (ITS) (Sigma Aldrich), 0.5% dimethyl sulfoxide (Sigma Aldrich), 2.5 ng/mL human epidermal growth factor (Life Technologies-Gibco), and 0.5 mg/mL gentamicin (Life Technologies-Gibco). The culture medium was changed every 3 days.

Limbal explants were kept in culture until a cellular outgrowth front of approximately 2 mm was present (1-2 weeks), and then the explants were removed to avoid stromal cell contamination and to allow further cell proliferation in the area previously occupied by the explant, as previously described [27]. Intermediate and final culture media were checked for sterility. The final product had to be negative for the tested microbiologic agents (aerobes, anaerobes, fungi, and mycobacteria) by using a validated test (Bact-ALERT) that inactivates antibiotics and thus eliminates the possibility of masked contamination. Additionally the final product had to be up to 80% confluent, which meant an average of 250,000 cells per product. All of these procedures, as well as the characterization of the cultures, were previously established in our regular cell culture laboratory [27] and then transferred to the GMP clean room. Further characterization of the cultures was not possible due to the scarceness of the tissues because of the limited amount of biopsy material. When cultivated cells were ready for CLET, the final cellular products were sent to the medical institution within 4 hr. of the surgery (Figure 1).

For limbal cells grown out of cadaveric explants, an additional culture was plated outside the GMP facility in parallel to the one planned for clinical use. In all cases, most cells (>70%) were highly positive for the limbal progenitor cell phenotype markers p63, CK15 and weakly positive for the corneal differentiated epithelium phenotype markers cytokeratins CK3/CK12 (data not shown). More in depth staining and complete characterizations were achieved before transferring this technique from the cell culture lab to the GMP clean room and thus not repeated [27, 28].

### 2.3. Surgical Procedures: Limbal Biopsy and CLET

All operations were performed by the same surgeon (coauthor José M. Herreras) and were carried out in a standard manner. Limbal biopsies were performed under topical anesthesia: 0.1% tetracaine chlorohydrate and 0.4% oxybuprocaine chlorohydrate solution (Colircusí Anestésico Doble, Alcon Laboratories, Ft. Worth, TX, USA), following the standards for aseptic procedures. Biopsies measuring 2 × 2 mm were obtained from regions of the corneoscleral limbus (Figure 1) where the palisades of Vogt were better defined by SLE and by IVCM at the initial visit [29].

For the CLET procedure, retrobulbar anesthesia was achieved with 3 cc of 5% lidocaine (Lidocaine Braun, Braun Medical SA, Melsungen, Germany). First, a conjunctival peritomy was performed and tissues were recessed leaving bare sclera. Fibrovascular pannus, if present, was scraped and removed from the recipient cornea extending to the limbal area, allowing a gentle 360° limbal peritomy to be performed. The scraped surface was polished with a diamond bur, and bleeding vessels were cauterized. Then the CLET graft was carefully lifted from the culture dish and placed with the epithelial limbal cells facing the recipient ocular surface so that cells were in immediate and close contact with the tissues (cornea and limbal areas) to be repaired. The graft was then sutured to the perilimbal episclera, 2–4 mm posterior to the limbus, with 8 interrupted 10-0 nylon stitches (Figure 1). Topical eyedrops (see below) were then applied and an 18–22 mm diameter bandage contact lens was set in place, and the eye was patched for 24 hr.

Twenty-four hours after surgery, each patient was evaluated and topical treatment with the fixed combination of 1% prednisolone acetate and 0.3% tobramycin (Tobradex, Alcon Laboratories) was prescribed 4 times per day for 6 weeks. Then, 1 mg/mL dexamethasone (Maxidex, Alcon Laboratories) was instilled 4 times a day and slowly tapered in the next 3 months. The contact lens was removed after complete disappearance of the amniotic membrane at 2 to 8 weeks, and the stitches were also removed at that time.

### 2.4. Evaluation Endpoints

Table 1 shows the summary of all evaluation endpoints performed at the initial visit and at 12 months for each of the 20 eyes subjected to CLET. At 24 and 36 months, only clinical evaluations were performed.

#### 2.4.1. Clinical Examination and Photographs

LSCD-related symptoms and the impact on each patient's life were evaluated with two self-administered questionnaires at the initial visit and 12 months after CLET. First, ocular surface-related symptoms caused by LSCD were evaluated with the ocular surface disease index (OSDI), where scores >12 indicate abnormal symptomatology, with >32 meaning severe symptoms [30]. Visual function-related aspects of the quality of life were evaluated with The National Eye Institute 25-Item Visual Function Questionnaire (NEI-VFQ-25) where higher scores on a scale 0 to 100 indicate better function [31].

Best corrected visual acuity (BCVA) was determined with a Snellen visual chart. Counting fingers, hand motion, light perception, and no light perception were converted to Snellen equivalents, 0.01, 0.001, 0.0001, and 0.00001, respectively, as published so that a mean value could be calculated [32]. One or more line changes in BCVA at 12 months after CLET were considered as improvement or worsening. However, BCVA improvement is never the primary goal of CLET, as this technique intends to reconstruct the corneal epithelium and will not necessarily affect stromal or endothelial pathologies for which other visual rehabilitation techniques may be needed after CLET. To avoid misinterpretation by the patient, the potential dependence of the visual prognosis on the surgical procedures (Table 2) judged to be necessary to restore vision after CLET was explained at the initial visit.

SLE (Topcon SL-8Z, Topcon Corp., Tokyo, Japan) with fluorescein corneal staining and photographs of the graft-treated eye were performed at each visit. In autografts, the biopsy site was also monitored. Two of the authors (Beatriz E. Ramírez and Margarita Calonge) evaluated the same parameters of LSCD independently, and in case of disagreement, the average score was recorded. Photographs were taken with diffuse light using the program IMAGENet (Fuji Fujifilm Finepix S1 Pro, Fuji Photo Film Co., Ltd., Tokyo, Japan). The following clinical characteristics were evaluated (Table 3): ciliary hyperemia, central corneal epithelial opacity, central corneal epithelial irregularity, corneal epithelial integrity including superficial punctate keratitis and persistent epithelial defects, and corneal superficial neovascularization based on the extension or area involved and neovessel length.

#### 2.4.2. In Vivo Confocal Microscopy

IVCM examination of the central cornea with the Heidelberg Retinal Tomograph HRT-3 and Rostock Cornea Module (HRT3, Heidelberg Engineering GmbH, Heidelberg, Germany) was performed at the initial visit and at 12 months after CLET to evaluate the phenotype of the epithelium covering the central cornea. Optical sections from the central cornea were taken at the basal and superficial layers of the epithelium. Basal cell morphology was classified as “corneal-like,” having regular, hexagonal cells with a cell diameter <20 *μ*m, or “conjunctival-like,” having closely packed round or irregularly shaped cells with a cell diameter of >20 *μ*m and occasional goblet cells. In some cases, the basal cell morphology was classified as “mixed,” with both phenotypes present [24, 33].

IVCM was also performed in the limbal area to select the site for biopsy in cases of autologous CLET. The area where more and better defined palisades of Vogt were found was selected as the site for biopsy. In some cases, the wounding and healing of the biopsy site were closely followed, as published elsewhere [29].

#### 2.4.3. Definition of an Overall Success, Partial Success, and Failure

As visual acuity by itself is a poor endpoint to evaluate the success of CLET and associated therapies, we made an integral evaluation of each patient through primary and secondary outcomes. Each patient was evaluated with this same protocol at 12 months after CLET.

*Primary outcomes* were as follows: (1) improvement in visual-related aspects of the quality of life evaluated with the NEI-VFQ-25 or improvement in ocular surface symptoms, evaluated by OSDI; (2) improvement by at least one step in at least 3 of the 4 following clinical parameters evaluated by SLE: ciliary hyperemia, central corneal epithelial irregularity, central corneal epithelial opacity, and superficial punctate keratitis; (3) complete absence (Grade 0) of persistent epithelial defects; and (4) presence of a more corneal-like phenotype in the central cornea as assessed by a change from the conjunctival phenotype to either corneal or mixed phenotype or from a mixed phenotype to a corneal phenotype as evaluated by IVCM [24, 33].

Secondary outcomes were as follows: (1) BCVA improvement of one line or more and (2) amelioration measured by at least a one-step decrease in the superficial corneal peripheral neovascularization area or neovessel length.

The outcome was considered successful only when all four primary outcomes were achieved. The outcome was considered partially successful when the patient presented only two of the primary outcomes or when one primary outcome in addition to one secondary outcome was achieved. Failure meant that only one or none of the primary outcomes were met.

After the first year, patients were evaluated only clinically by SLE every 6 months, until the end of follow-up, 3 years after CLET.

### 2.5. Statistical Analysis

Quantitative characteristics were expressed as means ± standard deviations (SD), and qualitative variables were described in percentages. The median and interquartile ranges (IQR) were used to summarize distributions of ordinal variables. Normality assumptions were checked by the Shapiro-Wilk test. The Wilcoxon signed-rank test was used to evaluate improvement of the subjective questionnaires OSDI and NEI-VFQ-25 and the change in quantitative and ordinal clinical variables at 12 months after CLET. Differences between the means of two independent groups were tested by Student's *t*-test or the nonparametric alternative, Mann-Whitney *U* test, if the normality assumption was not valid. Relationships between two qualitative variables were evaluated by Fisher's exact test. To compare the success rates for the three predefined prognostic groups and the type of CLET, the test for equality of proportions was used. Kaplan-Meier survival analysis was applied to estimate transplant survival. The log-rank test was used to compare the univariate survival curves of autologous and allogeneic type. Statistical analysis was performed using R Statistical Software version 3.1.0 (Foundation for Statistical Computing, Vienna, Austria) by a licensed statistician (coauthor IF).

## 3. Results

The demographic data and initial and final clinical data collected at 12 months for each case are presented in Table 1. CLET was performed in 20 eyes (12 males, 8 females) of 19 patients (age, 51.6 ± 14.5 years; range, 27–79 years). There was no significant difference in age between males and females (*t*-test, *p* = 0.3039). All cases were followed up to 3 years, although follow-up of Case 1 logically ended when he had a second CLET at month 13 (Table 1).

The three prognostic groups had the following etiologies: Group 1, composed of chemical injuries, had 7 eyes (35% of cases, 4 autografts, 3 allografts); Group 2, composed of immune-based inflammatory diseases, had 4 eyes (20%, 3 with Stevens-Johnson syndrome, 1 with mucous membrane pemphigoid, all allografts); and Group 3, composed of noninflammatory diseases, had 9 eyes (45%, 4 with sequelae from multiple surgeries, 3 with postinfectious keratitis, and 2 with congenital aniridia; 7 autografts, 2 allografts). Twelve eyes had total LSCD and 8 had severe LSCD. The time between disease onset and CLET was 77.2 ± 88.9 months (range, 6–321 months).

The time required for limbal cell expansion and cultivation on human amniotic membrane was 24.7 ± 5.8 days. Cultures from a cadaveric source required 26.1 ± 6.0 days, and cultures from an autologous source required 23.5 ± 5.7 days; however, this difference was not statistically significant (*t*-test, *p* = 0.4251).

Eleven eyes (55%) with unilateral disease had autologous CLET. Four cases belonged to prognostic Group 1, and 7 cases were in Group 3. The remaining 9 eyes (45%) received allografts: 3 eyes in Group 1, 4 eyes in Group 2, and 2 eyes in Group 3 (Table 2). There was no significant difference in the ages of the autologous and allogeneic recipients (*t*-test, *p* = 0.4929) or gender distribution (Fisher's exact test, *p* = 0.3618). Of the 9 eyes that received allogeneic CLET, two had unilateral disease. Case 18 had unilateral chemical injury but also had bilateral perennial allergic conjunctivitis, and for this reason we did not use her contralateral eye as donor. Another allograft with unilateral disease was Case 16, who like Case 1 had unilateral chemical injury. He initially received an autologous CLET; however, after the 12-month mandatory follow-up, he received an allogeneic CLET so as not to compromise the healthy eye with a second biopsy. Four eyes in Group 2 had allografts (immune-based diseases are always bilateral) and most cases in Group 3 (7 of 9) had autografts. Thus there was no independence between the source of transplanted cells (autografts versus allografts) and the etiology of the disease (Fisher's exact test, *p* = 0.0468).

There were no intraoperative or postoperative complications during either biopsy harvesting or CLET. No episodes of immune rejection were recorded in the eyes that had received allografts. All biopsies were extracted from the superior limbal area as the palisades of Vogt in this area are the richest source of limbal stem cells [2, 5, 8, 29].

Oral immunosuppression was used in all 9 allograft patients because immune rejection has been reported to occur in 23.8% of cases in a series of allogeneic CLET who were nonimmunosuppressed even though the patients were given high doses of systemic steroids [26]. Mycophenolate mofetil (1.5–2 g/day) was used in Cases 5 and 6; cyclosporine A (3–5 mg/kg/day) was prescribed in Cases 9, 10, 16, 17, and 18; and azathioprine (1.5–2 mg/kg/day) was used in Cases 15 and 19. The drugs were well tolerated in all cases, and no withdrawals or discontinuations were necessary. Mycophenolate mofetil had to be lowered from 2 g/day to 1.5 g/day in Case 5 due to asthenia; cyclosporine A was also lowered from 5 to 3 mg/kg/day in Cases 17 and 18 due to mild elevation in blood pressure. There were no episodes of graft rejection.

There was one adverse event. Case 1 developed a severe infectious conjunctivitis (*Staphylococcus aureus*, culture positive) 9 weeks after surgery. Although the infectious process was successfully controlled with topical antibiotics, the autograft started to deteriorate and was considered a failure at month 3. Due to the nature of this patient's job, we had recommended to him that he take extreme eye protective measures. He was noncompliant in this recommendation, which created a risk factor that probably resulted in the eye infection.

### 3.1. Overall Success/Failure Rate and Survival Analysis

**Figure 2:**
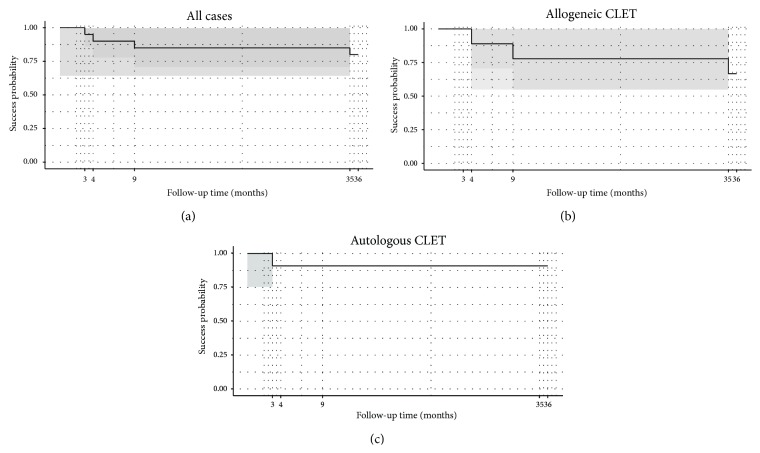
Kaplan-Meier survival (success) curves of the 20 cases undergoing cultivated limbal epithelial transplantation (CLET) according to follow-up time (maximum of 36 months) (a) and separated by the origin of cells, allogeneic CLET (b) and autologous CLET (c). Shaded areas represent the confidence bands. Survival analysis showed a probability of success at 1-2 years and at 3 years after CLET of 0.80 (confidence interval [CI] 95%, 0.643–0.996) and 0.75 (CI 95%, 0.582–0.966), respectively, for all cases (a). Allogeneic CLET had a 1-2-year and a 3-year survival probability of 0.667 (CI 95%, 0.420–1) and 0.556 (CI 95%, 0.31–1), respectively (b). The survival probability for autologous CLET was 0.9091 (CI 95%, 0.0867–0.7541) after 1, 2, or 3 years (c). The difference in survival between autografts and allografts was not significant (log-rank test, *p* value: 0.0949).

The overall success was 80% after both the one- and two-year follow-up periods. The rate decreased to 75% after 3 years. At 12 months after CLET, when we performed a thorough evaluation, 16 of the 20 eyes achieved the four primary outcomes (Table 1) while 4 eyes (20%, 3 complete failures, 1 partial success) were considered failures. All failures were from Group 1 (chemical injuries). At one year, the success rate was clearly lower in prognostic Group 1 (4 out of 7 eyes, 42.9%) than in Groups 2 and 3 (100%). Among these failures, Case 1 had autologous CLET, and Cases 16, 17, and 18 had allogeneic CLET. Therefore the success rate at one year for autografts was 90.9% (10 of 11), and for allografts it was 66.7% (6 of 9). There was no significant difference in the success/failure rates between the autologous and allogeneic CLET procedures (equality of proportions, *p* = 0.4315) (Figure 2).

**Figure 3:**
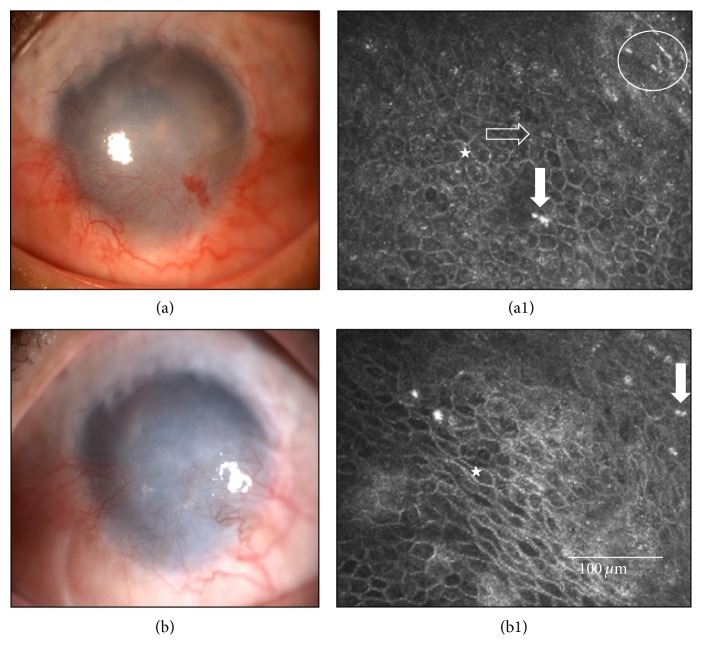
Case 17 (Table 1) before and after cultivated limbal epithelial transplantation (CLET). This 52-year-old male suffered a chemical injury in his right eye 5 years earlier. He developed total limbal stem cell deficiency and showed at the initial visit an opaque and vascularized cornea (a) with a conjunctival-like phenotype at in vivo confocal microscopy (IVCM) in central cornea (star), with goblet cells (horizontal arrow), inflammatory cells (vertical arrow), and Langerhans cell (circle), (a1). He received an allogeneic cultivated limbal epithelial transplantation (CLET). (b) After 12 months and although his symptoms and ciliary hyperemia had improved, this case was considered a CLET failure as his epithelial phenotype in central cornea (b1) was still conjunctival-like (star), with inflammatory cells (vertical arrow), evaluated by IVCM.

Even in the failed Cases 1, 17, and 18, there was subjective improvement as shown by lower OSDI scores and higher NEI-VFQ-25 scores (Table 1). Case 1 had another CLET (Case 16) in which the final outcome was graded as a partial success. This patient elected not to try to rehabilitate his chemically injured right eye any further as he remained mostly asymptomatic, and his left eye had full vision. Failed Case 17 (Figure 3) also chose not to do any additional procedure.

Case 18 failed after 9 months. After completing the 3-year follow-up, she entered a clinical trial on cell therapy that has finished recruitment (https://www.clinicaltrials.gov/ Identifier: NCT01562002).

All 3 failures and the partial failure after 12 months belonged to prognostic etiology Group 1. The success rate of this group, 42.9%, was significantly lower than for Group 2, the autoimmune-based cases (100% success, equality of proportions, *p* = 0.0096), or Group 3, the miscellaneous group (100% success, equality of proportions, *p* = 0.0096). None of the 3 failed cases in Group 1 deteriorated any further after the CLET failure.

Only one eye failed after the first year (month 35) after CLET (Case 5, Stevens-Johnson syndrome, prognostic Group 2), showing recurrence of epithelial barrier breakdown. This patient was also recruited at month 37 for the cell therapy clinical trial mentioned above.

Survival curve analysis (Figure 2) showed a probability of success (Kaplan-Meier) at 1 year and 3 years after CLET of 0.80 (confidence interval [CI] 95%, 0.643–0.996) and 0.75 (CI 95%, 0.582–0.966), respectively, for all cases. Autologous CLET had the same survival probability, 0.9091 (CI 95%, 0.0867–0.7541), after both 1 and 3 years. Allogeneic CLET had a 1- and 3-year survival probability of 0.667 (CI 95%, 0.420–1.00) and 0.556 (CI 95%, 0.31–1.00), respectively. The difference in survival between autografts and allografts was not significant (log-rank test, *p* = 0.0949).

### 3.2. Clinical Outcome

#### 3.2.1. Quality of Life and Ocular Surface Symptoms

The OSDI score for ocular surface symptoms at the initial evaluation was 49.5 ± 25.8 (Table 1), indicating that the symptoms were severe [30]. All cases showed a reduction in OSDI score following the first month after CLET (36.1 ± 25.2; Wilcoxon signed-rank test, *p* = 0.001). The improvement was maintained one year after CLET (34.3 ± 23.4, Wilcoxon signed-rank test, *p* = 0.0035; Table 1). The scores were not influenced by the source of donor cells (autologous versus allogeneic) (*t*-test, initial visit *p* = 0.0954, final visit *p* = 0.1420).

The visual-related quality of life score after CLET, measured by the NEI-VFQ-25, improved from 64.7 ± 22.9 at the initial evaluation to 71.5 ± 19.4 at the 1-year follow-up (Wilcoxon signed-rank test, *p* = 0.0057, Table 1).

Autograft cases started with significantly higher NEI-VFQ-25 values than allografts at the initial visit (76.9 ± 9.2 versus 49.8 ± 26.1, resp., *t*-test, *p* = 0.0147). The autograft cases also ended with significantly higher scores than the allograft cases at the final visit (81.5 ± 9.4 versus 59.3 ± 22.0, resp., *t*-test, *p* = 0.0176). However, when the increased percent of NEI-VFQ-25 score after CLET was compared for the two graft procedures, the difference between autografts and allografts was not significant (16.0 ± 20.0% versus 18.6 ± 26.0%, resp.; Mann-Whitney *U* test, *p* = 0.8788). Therefore, it is not possible to say that autografts improved visual-related quality of life issues more than allografts did.

#### 3.2.2. Parameters Evaluated by SLE

By SLE, complete reabsorption of the amniotic membrane was observed at 4 weeks after surgery in 12 eyes (60%), between 5 and 7 weeks in 7 eyes (35%), and in 8 weeks in one eye (5%).

Hyperemia was one of the signs that patients verbalized as having improved the most. It went from 3.0 ± 1.0 (median ± IQR) at the initial evaluation to 1.0 ± 1.3 at month 12 (Wilcoxon signed-rank test, *p* = 0.0001), improving in all 20 eyes (Table 1). At the initial visit, hyperemia was significantly greater (Mann-Whitney *U* test, *p* = 0.0170) in those patients that would later have allogeneic CLET, 3.0 ± 1.0, versus those who would receive an autograft, 2.0 ± 1.5.

The preoperative central corneal epithelial opacity score was 3.0 ± 1.3 (median ± IQR), and it improved significantly to 2.0 ± 1.3 (Wilcoxon signed-rank test, *p* = 0.0003) at one year (Table 1). For all three failed cases, the initial opacity score was at the maximum value of 4 and did not change after CLET (Table 1). It also remained unchanged in the partial success case (Case 16). For the remaining 16 eyes, central corneal epithelial opacity scores decreased. Considering only successful cases at the end of the 12 month follow-up, corneal central opacity was significantly greater in cases with autografts, 2.0 ± 1.0, compared to those with allografts (0.5 ± 1.0, Mann-Whitney *U* test, *p* = 0.0479).

Epithelial irregularity in the central cornea was significantly reduced from the initial score of 3.0 ± 1.0 to 1.0 ± 1.0 at the 12-month follow-up visit (Wilcoxon signed-rank test, *p* = 0.0002). For 17 of 20 eyes (85%), it decreased by a single step. For one eye, Case 13, it decreased by two steps. For the 3 cases that failed, the epithelial irregularity remained at the maximum pre-CLET level (Table 1). These results were independent of the source of cells (autologous or allogeneic) (Mann-Whitney *U* test, initial visit *p* = 0.2321, final visit *p* = 0.7419).

Superficial punctate keratitis, measured by corneal fluorescein staining, had a median pre-CLET score of 3.00 ± 1.25 that diminished at 12 months after CLET to 0.5 ± 1.0 (Wilcoxon signed-rank test, *p* = 0.0010). It increased in 2 of the 3 failed cases at 12 months and remained the same in the other failure and in the partially successful case. It was one of the parameters that improved in all successful cases, except for Case 13, where it was unchanged, as there was no initial corneal fluorescein staining (Table 1).

In the 12-month follow-up visit, corneal fluorescein staining was greater in those eyes that had received allografts, 1.0 ± 1.0, compared to those with autografts, 0.0 ± 0.5 (Mann-Whitney *U* test, *p* value = 0.0288). Considering only successful cases, there was tendency for greater staining in eyes that had received allografts compared to eyes that had received autografts, but the difference was not significant (Mann-Whitney *U* test, *p* value = 0.0814).

In agreement with the corneal staining, persistent epithelial defects also improved significantly (Wilcoxon signed-rank test, *p* = 0.0115). It was present in 8 cases before CLET and improved to total closure in successful cases. None of the failed cases had preoperative epithelial defects, however (Table 1). The source of cells, autografts versus allografts, did not affect this parameter (Mann-Whitney *U* test, initial visit *p* = 0.8965, at the final visit all cases equal 0).

Corneal neovascularization decreased in area from 3.5 ± 1.3 at the pre-CLET assessment to 2.0 ± 2.0 after 12 months (Wilcoxon signed-rank test, *p* = 0.0009) (Table 1). Similarly, the length decreased from 3.0 ± 2.0 to 2.0 ± 1.3 at the 12-month follow-up (Wilcoxon signed-rank test, *p* = 0.0043). For the three failed cases (Figure 3) and for the partially successful case, the values remained unchanged at 12 months from the initial high values. It also remained unchanged in Case 5 that failed at 35 months after CLET. Case 20, although successful, had mild neovascularization that remained unchanged in area but increased one step regarding neovessel length (Table 1). Corneal neovessel scores were unaffected by the nature of the cells (auto- or allogeneic) transplanted (Mann-Whitney *U* test, initial visit neovessel area *p* = 0.7427 and neovessel length, *p* = 0.66; final visit neovessel area *p* = 0.4589 and neovessel length *p* = 0.8099).

### 3.3. IVCM Determination of Epithelial Phenotypes in the Central Cornea

**Figure 4:**
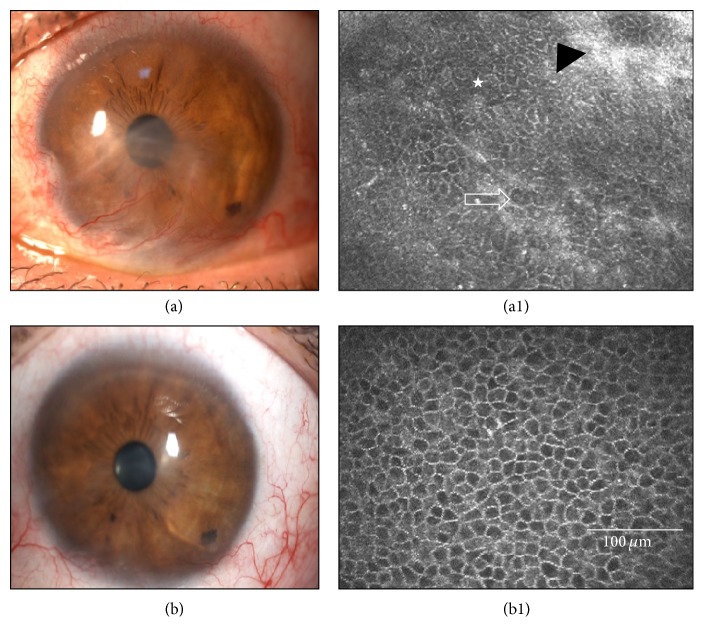
Case 8 (Table 1) before and after cultivated limbal epithelial transplantation (CLET). This 36-year-old man had limbal stem cell deficiency due to a unilateral chemical burn, with vascular pannus invading the visual axis (a). (a1) In vivo confocal microscopy (IVCM) shows a typical conjunctival-like epithelial phenotype in his central cornea (star), with goblet cells (arrow) and fibrosis (black arrowhead). This case was graded preoperatively in terms of visual prognosis as Grade 1, meaning that only cultivated epithelial transplantation (CLET) would be required for visual rehabilitation. After 12 months, this case was considered successful as all clinical signs improved (b) and symptoms decreased and IVCM showed an epithelial corneal phenotype (b1).

**Figure 5:**
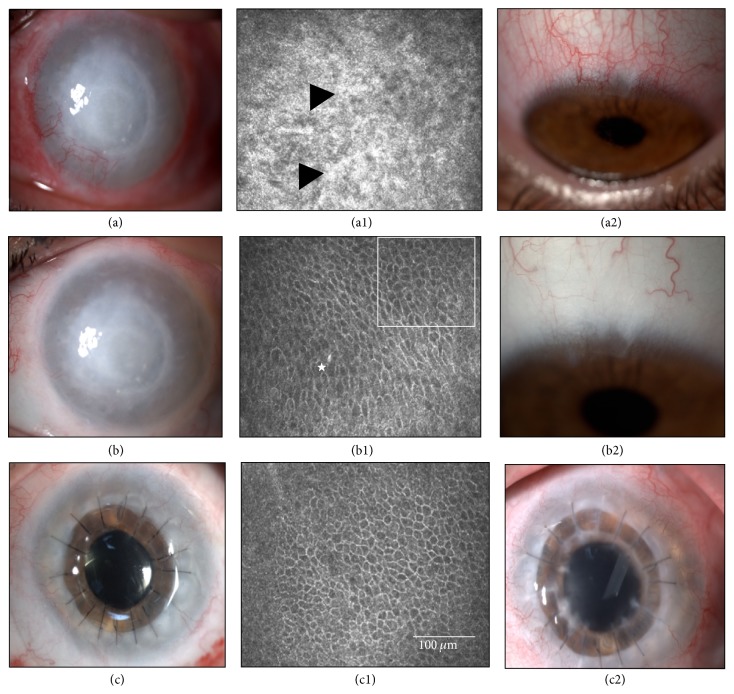
Case 11 (Table 1) before and after cultivated limbal epithelial transplantation (CLET). This 27-year-old male had a total limbal stem cell deficiency due to an early failed penetrating keratoplasty 7 years before. It was performed 5 years after a contact lens-related* Acanthamoeba* keratitis (a). (a1) In vivo confocal microscopy (IVCM) in the central cornea showed intense fibrosis (black arrows) and a conjunctival epithelial phenotype. (a2) Limbal cells for cultivated limbal epithelial transplantation (CLET) were obtained from his contralateral healthy eye, the biopsy site of which is shown 3 months after biopsy. (b) Twelve months after autologous CLET, corneal neovascularization had almost vanished and IVCM showed a mixed epithelium phenotype (b1), conjunctival phenotype (star), and corneal phenotype (square). (b2) Limbal donor site 12 months after biopsy. (c) Fourteen months after CLET, a penetrating keratoplasty and cataract removal were performed, followed 12 months later with a compact and clear graft. IVCM showed a corneal phenotype (c1). (c2) The corneal transplant was still successful after 2 years (3 years after CLET) although an Ahmed valve was implanted 10 months after corneal transplant to treat his elevated intraocular pressure.

Evaluation of the central corneal epithelial cell phenotypes by laser IVCM was the most objective primary endpoint [24, 33]. Before CLET, 13 eyes (65%; Cases 3, 4, 8, 9–17, and 20) had a conjunctival epithelial phenotype in the central cornea (Figures 3–5). One year after CLET, 6 of the 13 cases (Cases 3, 8–10, 13, and 20) improved to the corneal-like epithelium phenotype (Figure 4), and 5 eyes (Cases 4, 11, 12, 14, and 15) evolved to a mixed phenotype (Figure 5). The partially successful case (Case 17) and one of the 3 failed cases (Case 17) maintained the conjunctival phenotype. The remaining 7 eyes (Cases 1, 2, 5–7, 18, and 19) were classified in the initial examination as having the mixed epithelium phenotype. One year after CLET, 5 of these eyes (71.43%) changed to the corneal phenotype (Cases 2, 5–7, and 19). The 2 remaining cases (Cases 1 and 18) worsened to the conjunctival phenotype and were consequently considered failures. The type of transplant, autograft or allograft, had no influence in these results (Fisher's exact test, initial visit *p* = 0.6424, final visit *p* = 0.3359).

In summary, at the end of the first year after CLET, 80% of the cases had improved epithelial status in the central cornea. Of these cases, 68.8% improved from conjunctival to corneal phenotype. Of the total number of cases, the epithelial status of 10% remained unchanged and 10% worsened.

### 3.4. BCVA, Visual Potential, and Visual Rehabilitation

In successful cases, BCVA increased from 0.15 ± 0.24 at the initial visit to 0.25 ± 0.33 at 12 months after CLET (Wilcoxon signed-rank test, *p* = 0.0059). However, when all cases were analyzed, the increase in BCVA, from 0.15 ± 0.25 initially to 0.20 ± 0.25 at 12 months, was not significant (Wilcoxon signed-rank test, *p* = 0.0914). These results were not affected by the source of donor cells (autologous or allogeneic CLET) (Mann-Whitney test, initial visit *p* = 0.1779, final visit *p* = 0.2022).

In 10 successful cases at one year after CLET (Cases 5, 8–10, 12–15, 19, and 20), BCVA improved one line or more (Table 1). These cases represented 50% of the total 20 eyes and 62.5% of the 16 successful eyes. These 16 eyes were the only ones with any probability to improve (Table 2, prognostic Grade 1, 2, 3, or 4). The visual prognosis was Grade 0 in the remaining 4 eyes for which the retinal pathology was irreversible. Five of the 10 cases (Cases 5, 8, 9, 10, and 20) were previously assigned a visual prognosis of Grade 1, meaning that CLET alone, without further intervention, was likely to improve vision (Table 2, Figure 4). Two more of those 10 eyes (Cases 12 and 13) were assigned Grade 3, meaning that a corneal transplant would be needed. Unexpectedly, they improved sufficiently to refuse any further rehabilitation. The predictions in Cases 11 (Figure 5) and 14, Grade 4, were accurate. Case 14 required a full thickness corneal transplant and cataract removal. Although the patient was very satisfied with the result, only a modest gain in BCVA was achieved. This patient had been warned in advance about the potential for a poor visual result regardless of any rehabilitation. She had undergone three previous surgeries due to retinal detachment, and the affected eye had 45-degree exotropia before CLET. Case 15 decided not to have any further rehabilitation as he had gained comfort, and due to moderate to severe nystagmus, it was unlikely that his vision would improve. The visual potential prediction in Case 19 was also adequate with an improved BCVA of 0.2 with a mild nystagmus.

BCVA remained unchanged in 8 cases (Cases 2–4, 6, 7, 11, 16, and 17; 40% of the total 20 eyes and 37.5% of the 16 successful eyes). Four of the successful eyes (Cases 2, 3, 4, and 7) were given a visual potential of Grade 0, as all of them were previously affected by irreversible retinal pathologies. Thus no change in BCVA was expected. The central cornea status of successful Case 6 (visual potential Grade 1) improved, but his already good BCVA did not improve, which was attributed to cataract progression. In one more successful eye (Case 11, Figure 5), as predicted based on the assigned visual potential of Grade 4, BCVA only improved from hand motion to 0.4 after corneal transplantation and cataract removal. Partially successful Case 16 had a predicted visual potential of Grade 3, and he refused to pursue a corneal transplant because of the improved comfort after CLET and the full vision with his fellow eye. Case 17 (Figure 3), with a predicted visual potential of Grade 4, failed, and no further action was considered.

Vision became further impaired in 2 of the 3 cases (Cases 1 and 18) considered as failures. This represents 10% of the total 20 eyes. In Case 18, BCVA diminished dramatically, not because her corneal status became further impaired (Table 1), but rather due to intense progression in her incipient cataract. The rapid development of the cataract could have been due to the large amount of steroids that she needed, because in addition to eyedrops, she also required both inhaled and oral steroids for her severe asthma. This patient subsequently enrolled in the ocular surface cell therapy clinical trial previously mentioned.

## 4. Discussion

With this study, we have added to the existing body of data that supports the safety and efficacy of CLET procedures performed under GMP rules for the management of ocular surface failure due to LSCD. Overall, our major findings are that (1) 80% of the cases were successful at 1 and 2 years after transplantation and 75% remained successful at 3 years, (2) the efficacies of autologous and allogeneic limbal cell culture were similar to one another, and (3) among the three prognostic groups, those with chemical burns had the least satisfactory outcomes.

We also showed the feasibility of mild immunosuppression for a year. There were no adverse effects and no episodes of immune rejection [26]. Whether or not immunosuppression is absolutely necessary is yet controversial due to the reported immunoprotective properties of limbal stem cells against inflammatory challenges of the ocular surface [3, 22].

This study also clarifies the need for strict and well-predefined success criteria, which has been stressed by almost all authors working in this field. We adopted a modified combination of the criteria used by Shortt et al. [24]. These or a different arrangement of evaluation outcomes should be agreed upon so that studies from different parts of the world could be more comparable and so that multicenter studies and clinical trials could be a reality [34].

The intention of CLET is to improve the damaged or diseased corneal epithelium phenotype and integrity and subsequently to improve corneal barrier function. In support of that goal, this study also confirms laser IVCM as an excellent minimally invasive imaging technique to visualize with great detail the quality of the corneal epithelium before and after CLET. Although it may not be necessary in routine clinic evaluations, it proved to be a powerful tool in the evaluation of the corneal epithelium in our study and in clinical trials where objective endpoints are required [24, 33].

Since the first two patients undergoing successful autologous CLET for unilateral chemical burns in 1997 [21], many more reports have been published using this transplantation technique. Not surprisingly, these reports have utilized different sets of patients, cell product preparation protocols, surgical techniques, evaluation criteria endpoints, and follow-up periods. There are many relevant reviews focused on different aspects of corneal stem cell transplantation that are beyond the scope of this publication, which focuses mainly on clinical reports. In general, CLET success rates are above 60% as reviewed by Baylis et al. in 2013 [6] and earlier by Shortt et al. in 2007 [35].

Our study can be better compared to those performed, as ours was, after the EU Tissues and Cells Directive and GMP rules became mandatory in the EU in 2006. In our series of 20 cases, the success rate was 80% at both 1 and 2 years and 75% at 3 years. Nevertheless, the obvious differences in patients and protocols prevent full and totally reliable comparisons among different studies.

The first study published in Europe in compliance with European regulations and GMP rules was by Shortt et al. in 2008 [24] (Moorfields Eye Hospital-University College of London, London, United Kingdom). They reported 10 cases with 6-month success of 60%, evaluated clinically by corneal impression cytology and by IVCM. They had the same proportion of chemically injured eyes, 40%, as our series, with no cases of immune-based cicatrizing ocular surface diseases. In contrast, 20% of our cases were immune-based cicatrizing diseases. Their series, as ours, included both autograft and allogeneic CLET cases. Although no statistical analyses were done, these authors reported better results for allografts (70%, 7 of 10 cases) than for autografts (33%, 3 of 10 eyes). We had a slightly better prognosis for autografts but the difference in outcomes between autografts and allografts was not statistically significant. The fact that their allograft tissues derived from donor cadavers were larger than the autologous tissue might have influenced their results. In contrast, we used the same amount of tissue for each type of transplant. However, it is difficult to say if the amount of tissue starting for the in vitro expansion could have a significant effect on the outcome. Vision improved in 70% of Shortt et al.'s, 10 patients, whereas it did so in 50% of our patients. In the subset of our cases that were considered successful, 62.5% of the eyes showed vision improvement.

Preparation of the cell product by Shortt et al. [24] was similar to ours. They also used amniotic membrane but prepared the limbal cells in a suspension culture system, while we used an explant culture system. We have ample experience in both techniques and are now considering changing to the cell suspension technique. Currently in our laboratory, cell suspensions achieve more confluent primary cell cultures, in less time, with more cells and with less fibroblast contamination, although both techniques have the same cell viability and proliferative capacity and same electron microscopy characteristics (unpublished data). These results, however, are derived from expanded cadaveric tissues sent for research purposes and thus are not suitable for clinical uses. The tissues were from very old donors (around 80 years old) and had longer elapsed times between enucleation and culture (data not shown). It is possible that the differences between cell suspension- and explant-derived cells will not be the same with the cadaveric tissues used for clinical purposes. Cadaveric tissues suitable for clinical applications are always derived from donors who are younger in age (less than 60 years old in our series) and for which there is less time between enucleation and usage. These factors are reported to be influential in clinical outcomes [36]. In contrast to cadaveric tissues, autologous tissues are always fresher (processed within 4 hr. in our cases) and not available for research. Shimazaki et al. compared the explant technique without the use of feeder cells or air lifting to the explant techniques with 3T3 feeder cells and airlifting and with the cell suspension technique [37]. They found similar in vitro characteristics among the culture techniques, but the clinical results were best with cells derived by suspension culture, in agreement with other authors [21, 25]. This is one of the many aspects that needs careful evaluation in future well-designed studies.

The surgical technique and medical management used by Shortt et al. [24] were quite similar to ours, although they placed their cell product with cells facing externally, while we did just the opposite. These authors also relied on IVCM as the primary objective measure of success, and in fact we followed their parameters. We decided not to perform impression cytology, because in agreement with other authors [25, 38], it is painful, unnecessary, and risky. Impression cytology requires stripping off 3-4 layers of corneal epithelium, a procedure that unnecessarily stresses the new, healthier tissue. Further, impression cytology does not add any relevant information to the clinical diagnosis [38]. IVCM is better and more safely serves as an objective method to evaluate the corneal epithelium [24, 33]. In any case, neither IVCM nor other more invasive procedures are necessary to establish the success or failure of CLET in the routine clinical environment.

Shortt et al. recently reported the 3-year results on LSCD for Stevens-Johnson syndrome and congenital aniridia [34], diseases that we have classified in prognostic Groups 2 and 3, respectively. They found positive results at one year and a deterioration of the tissues thereafter. Our two congenital aniridia cases and two of the three Stevens-Johnson cases, however, remained stable. The third Stevens-Johnson case failed at 34 months. Immune-based cicatrizing disorders are very difficult to treat, and they have a poor prognosis [37]. Although most authors have claimed stability after one year, this is not yet fully known, especially with respect to the different etiologies. There are no published large case series on these nonchemical burn eyes as they are rare diseases. Our results will need corroboration as it would be very useful to know how the duration of CLET success depends on the etiology of each disease.

Two years after the first European study by Shortt et al. [24] that followed the institution of GMP regulations, Kolli et al. [39] (Royal Victoria Infirmary-Newcastle University, Newcastle Upon Tyne, United Kingdom) attempted to attribute the success or failure of treating LSCD with CLET solely to the cell product. They recruited a strictly uniform group of 8 patients with unilateral total LSCD due to chemical burns. They treated the cases by autologous CLET, using amniotic membrane and xeno-free products under GMP rules. Based on clinical evaluation, they reported a 100% success rate with a mean follow-up of 19 months. Vision improved in 5 of the 8 eyes, a success rate similar to our own (62.5%). In our study, we had only 4 chemical burn cases treated with autologous CLET (Cases 1, 8, 13, and 20, Table 2), which were the most comparable with Kolli et al. [39]. While one case failed, vision improved in the other 3 cases.

Using fibrin-cultured autologous CLET, Rama et al. [25] reported on 107 cases followed up for a mean of about 3 years. Their success rate was 68.2% after one graft, with a final successful clinical outcome of 76.6% after regrafting 11 eyes. This success rate is very similar to ours, 75% at 3 years, even though most of their cases were chemical/thermal injuries and they only performed autografts. Full evaluations were done at 1 year, as we did, and grafts that were considered successful remained stable. Failed cases did not worsen as compared with baseline, as we also observed. Their judgment for success/failure was based on clinical grounds, and like us, they stopped doing corneal impression cytology for the same reasons that we did not do them.

The main difference with our protocol and that of Rama et al. is the stem cell final product. They used a fibrin-derived matrix instead of amniotic membrane like we used. Additionally, Rama et al. used clinical grade-certified 3T3-J2 cells, a mouse embryonic 3T3 fibroblast cell line that was lethally irradiated, as a feeder layer in contrast to our use of denuded amniotic membrane. In contrast to our use of the explant technique and the subsequent outgrowth of primary cell cultures, they used primary cell cultures that were trypsinized to transfer the cells to the fibrin-based substrate. Additionally, their cell cultures were transplanted 24–36 hr. after transfer to the transport container while ours were used within the next 4 hr. In summary, different etiologies, different cell sources (always autologous for Rama et al.), and different cell product protocols prevent reliable comparisons. Nevertheless, despite all the significant differences, our final success rates were fairly similar. This perhaps indicates that different protocols may work equally well as long as viable, stem cell-like cells are transplanted, as they showed in their series. How all of the possible variables affect the final outcome remains unknown, and it would be extremely difficult to test each of the variables independently. Thus we advocate reaching an agreement on all possible variables in all centers willing to participate in multicenter studies.

In 2010, Pauklin et al. [40] (University of Duisburg-Essen, Essen, Germany) published a series of LSCD cases for 32 total CLET eyes and 12 partial ones. The mean follow-up time was approximately 2 years, and their success rate, based on clinical grounds, was similar to ours, between 68% (full corneal stability) and 84% (clear central cornea). Grafting was significantly more successful in eyes treated with autologous CLET (77% of 30 eyes) than with allogeneic CLET (50% of 14 eyes, cadaveric or living related donors), in disagreement with our results and others [24, 41]. Perhaps the better prognosis of the etiologies undergoing autologous CLET could explain their better results. While they did not state that they adhered to the GMP standards, their cell product was fairly similar to ours, although they used intact amniotic membrane while we used denuded membranes. It seems as though they must have applied the cells facing externally because they placed a second amniotic membrane as a patch. Interestingly, they provoked a pharmacological eyelid ptosis to protect the graft, while we used a large contact lens.

In 2011, Sangwan and his group [42] (L. V. Prasad Eye Institute, Hyderabad, India) published a 10-year (2001–2010) retrospective study on xeno-free autologous CLET, including the largest series reported, 200 unilateral total LSCD cases due to chemical burns. Their cell product, prepared without clear reference to GMP adherence, was a limbal cell monolayer on denuded amniotic membrane, as ours; however, they did not plate the whole explant, but rather, shredded it first to obtain primary cultures. They reported 71% success rate, evaluated on clinical grounds, with a mean follow-up of 3 years, very similar to our series and as reported by most authors. Failures occurred mainly within the first year. They also reported a visual gain of two lines in 60.5% of eyes with no further surgery, similar to our 50–62.5% with visual gain. They used sutures or fibrin glue and did concomitant symblepharon surgery in 45% of cases and keratoplasty in 5% of them. Although understandable in clinical practice, performing more than one surgery certainly increases postoperative inflammation, and that can affect limbal cell survival. In fact, the same group later reported a worse prognosis when penetrating keratoplasty was performed at the same time as autologous CLET than when CLET was done first and keratoplasty at least 6 weeks after [43]. We waited a year to schedule our two penetrating keratoplasty cases, as did most authors, including Basu et al., who later waited at least one year after CLET before the next surgery [44]. We strongly recommend avoiding any surgery other than CLET in the context of prospective clinical studies and trials so as not to mask results from CLET.

These authors also reported similar results with autologous CLET irrespective of whether the limbal biopsy was taken from a healthy section of the affected eye or from the contralateral eye [45]. Several groups have reported that CLET can be safely repeated [25, 42, 46, 47]. We repeated one case in our series that ended up as partial success (Case 16), but with great alleviation of symptoms.

Sejpal et al. [47] also have significant experience in CLET for pediatric patients, mostly due to chemical/thermal injuries. The success rates for these cases were similar to those reported for adults. Similarly good results for pediatric CLET in 26 ocular burn cases were reported by Vajpayee et al. [48]. We had no pediatric cases, as the main cause for LSCD in children, chemical injuries, is exceedingly rare in our geographic area.

Prabhasawat et al. [41] (Siriraj Hospital-Mahidol University, Bangkok, Thailand) published a series of 19 LSCD cases (13 total, 6 partial) managed with CLET. Whether or not GMP rules were followed was not stated. They used clinical observations and impression cytology to evaluate success/failure, and their series had etiologies similar to ours. They reported final success of 73.7% with a mean follow-up of 26 months, although they included some cases followed up for only 6 months. They used limbal sections ranging from 2 × 2 mm to 2 × 2 cm from contralateral eyes. The epithelium was detached from the limbal explants with dispase and seeded on amniotic membrane with a final yield of 2–4 layers of epithelium. This suggests that limbal cells may have been differentiated as what happens when stem cells become multilayered [49]. In two cases at the time of surgery, a 6-mm central disk was punched out of the amniotic membrane upon which the cells were cultured, which would have eliminated a significant number of transplanted cells. They also removed symblepharon in some patients at the same time of grafting, even using mitomycin C, which is unusual and can mask results. They stated that the autografts were less successful (66.7%, 8 of 12) than the allografts (85.7%, 6 of 7). These results were similar to Shortt et al. [24] but contrary to Paulkin et al. [40] and us. Perhaps the fact that 83.3% of their autografts and 42.9% of their allografts were for chemical burns could explain this difference. Additionally, they had more symblepharon cases within the eyes that had received autografts. While the final success rate was similar to ours, differences in surgical management and cell products make comparisons difficult.

In 2013, Qi et al. [26] (Shandong Eye Institute, Qingdao, China) published, without any statement on GMP policy, a series of 42 eyes undergoing allogeneic CLET. They centered their work on the incidence of immune rejection, describing the clinical characteristics. They reported 23.8% immune rejections, all in eyes with chemical/thermal burns and occurring between 1 and 6 months. The outcome for the remaining 32 eyes (76.2%) was successful. This success rate was similar to ours, although our failures were not immune rejection-related, probably because our patients had systemic immunosuppression. This immune rejection rate cannot be taken as the rate under nonmedicated circumstances, as their patients received high, immunosuppressive doses of systemic steroids. They also had frequent use of topical steroids and 1% cyclosporine eyedrops. We, as others, strongly prefer using oral nonsteroidal immunosuppression rather than high doses of oral steroids that can produce a high frequency of undesirable and severe side effects.

The most recent series reported in the EU was in 2014 by Zakaria et al. [38] (Antwerp University Hospital, Antwerp, Belgium) in which 15 patients had total and 3 had partial LSCD, with etiologies similar to ours. Autologous (*n* = 15) or allogeneic (*n* = 3, 2 from HLA-matched living related donors and 1 from cadaveric donor) xeno-free CLET was performed and followed up for 22 months. The clinically judged overall success rate was 67%, worse in chemical burns but similar to our 75% at 3 years. Contrary to our results, these authors did not see a significant reduction in pain or photophobia. This could be due to the great difficulty in properly assessing clinical symptoms in patients and/or the fact that they used clinical scales for pain and photophobia to measure symptoms, while we used the OSDI and NEI-VF-25 assessments. They cultivated explants on top of amniotic membranes and followed the same technique as ours to prepare the host bed. However, they applied the composite graft, for example, amniotic membrane and cells facing up, with tissue fibrin glue. They then placed a second amniotic membrane on top and tucked it under the conjunctiva before suturing it. A big difference is that, at the time of surgery, the composite graft contained both the cultured cells and the original limbal biopsy, which we removed when outgrowth was seen. They do not state if they operated under GMP rules, although they probably did as it is mandatory in the EU. They started offering corneal transplantation one year post-op, as we did. We also agree with these authors in not performing corneal impression cytology because the cell pick-up was low and the procedure risked disrupting the transplanted epithelium.

## 5. Conclusion

In conclusion, we showed that limbal cell expansion and culture can be successfully achieved following our protocol using GMP conditions and following EU regulations. Both autologous and allogeneic CLET significantly improved the quality of corneal epithelium in patients with ocular surface failure due to LSCD. It enabled subsequent improvement in symptoms, increasing the quality of life in 75% of the patients after 3 years. These results confirm other reports that CLET is a successful treatment for ocular surface failure due to LSCD, although in some patients it seems to be insufficient. The merits of having a predefined prognostic schema like ours (Table 2) or one similar to it seem self-evident. It provides documentable guidelines for different surgical and medical treatments and sets reasonable physician and patient expectations for outcomes based on the current status of the eye. Thus we encourage the routine use of it or ones like it. In our study, visual improvement was achieved in those eyes previously classified in our schema as having the potential to gain vision with only CLET. Symptoms were greatly alleviated in all successful patients. Our study also affirms that laser IVCM is a good, minimally invasive, technique to assess the main evaluation endpoint, corneal epithelium restoration in the central cornea, in clinical studies and trials. Finally, we demonstrated that a strict but ample clinical composite score coupled with an objective imaging technique is ideal tool for use in future clinical trials.

Finally, consensus in cell preparation protocols and patient-related issues (pre-, intra-, and postoperative) should be sought to coordinate multicenter clinical studies and trials that help answer many of the still remaining questions about this otherwise overall successful transplantation technique. Needless to say, clinical efforts must be paralleled with research efforts so that these complex and difficult blinding diseases can be better treated.

## Figures and Tables

**Table 1:** Preoperative data and outcomes at 12 months of 20 eyes with ocular surface failure due to limbal stem cell deficiency syndrome (LSCD) subjected to cultivated limbal epithelial transplantation (CLET).

Eye number/gender/age	LSCD etiology/group^*∗*^/grade^†^	Cell source	OSDI I/F	NEI-VFQ-25I/F	Visual potential^‡^	BCVA I/F	Ciliary hyperemia (0–4) I/F	Central corneal epithelial opacity (0–4) I/F	Corneal neovessels area (0–4) I/F	Corneal neovessels length (0–4) I/F	Central corneal epithelial irregularity (0–3) I/F	SPK (Fluor) (0–5) I/F	PED (0–4) I/F	Epithelial phenotype (IVCM) I/F	One-year final outcome *Comments *
1/M/47	Chemical injury/1/T	Auto-	77.0/39.5	68.8/68.8	4	0.05/0.001	3/2	4/4	4/4	3/4	3/3	0/2	0/0	Mixed/Conj	**Failure month 3** *2nd CLET (number16) *
2/F/71	Multiple surgeries/3/S	Auto-	45.0/25.0	71.4/74.5	0	0.00001/0.00001	3/0	1/0	3/0	2/0	3/1	3/1	1/0	Mixed/corneal	**Success**
3/F/79	Multiple surgeries/3/T	Auto-	28.0/20.0	68.8/74.4	0	0.00001/0.00001	1/0	3/2	3/1	2/1	3/1	4/0	0/0	Conj/corneal	**Success**
4/M/69	Multiple surgeries/3/T	Auto-	44.0/9.4	87.8/89.7	0	0.00001/0.00001	2/0	3/2	4/2	3/2	2/1	3/0	2/0	Conj/mixed	**Success**
5/F/63	Stevens Johnson/2/S	Allo-	65.0/50.0	71.6/71.6	1	0.05/0.06	3/1	2/0	2/2	2/2	3/1	3/1	1/0	Mixed/corneal	**Success** ***Failure month 35***
6/M/62	MMP/2/S	Allo-	4.1/0.3	91.9/91.9	1	0.8/0.8	3/0	1/0	4/0	4/0	2/1	3/0	0/0	Mixed/corneal	**Success**
7/M/66	Multiple surgeries/3/S	Auto-	47.0/67.8	81.2/84.2	0	0.001/0.001	3/1	3/2	4/2	4/3	3/2	3/0	1/0	Mixed/corneal	**Success**
8/M/36	Chemical injury/1/S	Auto-	31.0/29.1	80.6/80.6	1	0.25/0.6	2/1	3/1	2/1	4/2	3/1	2/0	0/0	Conj/corneal	**Success**
9/F/34	Stevens Johnson/2/T	Allo-	98.0/89.5	40.7/57.4	1	0.05/0.2	3/2	2/1	1/0	1/1	2/1	4/1	1/0	Conj/corneal	**Success**
10/F/63	Stevens Johnson/2/T	Allo-	78.0/30.0	52.1/76.9	1	0.5/0.8	3/2	2/0	4/3	3/1	2/1	2/1	1/0	Conj/corneal	**Success**
11/M/27	Postinfectious keratitis, PKP/3/T	Auto-	46.0/22.7	69.1/72.8	4	0.001/0.001–0.4 after 2nd surgery	4/1	4/3	3/1	2/1	3/2	2/0	0/0	Conj/mixed	**Success** *PKP + cataract (month14) + valve *
12/M/52	Postinfectious keratitis/3/T	Auto-	62.5/5.0	93.0/95.3	3	0.05/0.2	1/0	3/2	4/2	4/2	3/2	2/0	0/0	Conj/mixed	**Success** *Refused PKP *
13/M/36	Chemical injury/1/S	Auto-	8.3/8.3	79.6/74.7	3	0.2/0.5	1/0	3/1	2/1	3/2	3/1	0/0	0/0	Conj/corneal	**Success**
14/F/54	Postinfectious keratitis, PKP; RD surgeries/3/T	Auto-	28.0/18.8	81.9/84.7	4	0.0001/0.001–0.01 after 2nd surgery	2/1	4/2	4/3	3/2	2/1	4/1	0/0	Conj/mixed	**Success ** *PKP + cataract (month 30) *
15/M/37	Congenital aniridia/3/S	Allo-	98.0/56.8	16.2/28.1	4	0.001/0.01	3/1	3/2	3/2	3/2	3/1	4/1	2/0	Conj/mixed	**Success** *Refused further surgeries *
16/M/48	Chemical injury; previous CLET (number 1)/1/T	Allo-	33.0/22.7	66.6/64.3	3	0.001/0.001	3/2	3/3	4/4	4/4	2/1	2/2	0/0	Conj/Conj	**Partial success** *Refused PKP *
17/M/52	Chemical injury/1/T	Allo-	52.0/50.0	12.6/37.7	4	0.001/0.001	3/1	4/4	3/3	3/3	3/3	3/3	0/0	Conj/Conj	**Failure month 4**
18/F/33	Chemical injury, PKP, severe ocular allergy/1/T	Allo-	65.0/22.7	60.9/73.0	4	0.62/0.01	4/2	4/4	4/4	4/4	3/3	2/3	0/0	Mixed/Conj	**Failure month 9**
19/F/49	Congenital aniridia/3/T	Allo-	47.7/63.6	35.3/33.1	2	0.02/0.05	3/0	2/1	4/1	3/2	3/1	2/0	0/0	Mixed/corneal	**Success**
20/M/53	Chemical injury/1/S	Auto-	31.3/9.4	63.7/96.3	1	0.5/0.8	2/1	3/1	1/1	1/2	3/1	4/0	2/0	Conj/corneal	**Success**
Mean (SD)	—	—	49.5 (25.8)/34.3 (23.4)	64.7 (22.9)/71.5 (19.4)		0.15 (0.25)/0.20 (0.31)	—	—	—	—	—	—	—	—	—
Median (IQR)	—	—	—	—		.	3.0 (1.0)/1.0 (1.3)	3.0 (1.3)/2.0 (1.3)	3.5 (1.3)/2.0 (2.0)	3.0 (2.0)/2.0 (1.3)	3.0 (1.0)/1.0 (1.0)	3.0 (1.3)/0.5 (1.0)	0.0 (1.0)/0.0 (0.0)	—	—

^*∗*^Group 1: chemical injuries, Group 2: immune-based inflammatory diseases, Group 3: noninflammatory diseases; ^†^T: total, S: severe; ^‡^visual potential: 1, improvement with CLET only (corneal opacity was only superficial); 2, improvement with one surgery different from corneal transplant after CLET (i.e., cataract removal); 3, improvement with subsequent corneal transplant after CLET (corneal opacity was full thickness); 4, improvement with subsequent corneal transplant plus another surgery (cataract removal unless otherwise specified) after CLET, and 0: no possibility of improvement (i.e., due to irreversible retinal pathology). Auto-, autologous; Allo-, allogeneic; BCVA, best corrected visual acuity; BCVA values 0.01, 0.001, 0.0001, and 0.00001 equivalent to counting fingers, hand motion, light perception, and no light perception, respectively; Conj, conjunctival; Fluor, fluorescein staining; IQR, interquartile range; IVCM, in vivo confocal microscopy; MP, mucous membrane pemphigoid; NEI-VFQ-25, National Eye Institute-Visual Function Questionnaire; OSDI, ocular surface disease index; SPK, superficial punctate keratitis; PED, persistent epithelial defect; PKP, penetrating keratoplasty; and SD, standard deviation.

**Table 2:** Prognostic classification on the potential for visual recovery in patients suffering from limbal stem cell deficiency and scheduled for cultivated limbal epithelial transplantation (CLET).

Visual prognosis	Ocular media opacity	Surgeries judged to be necessary to recover full potential vision
Grade 1	Corneal opacity restricted to anterior cornea (epithelial and anterior stroma)	One surgical procedure: CLET only

Grade 2	Corneal opacity restricted to anterior cornea (as Grade 1) plus another noncorneal reason for visual loss (e.g., cataract)	Two surgical procedures: CLET+ noncorneal surgery (e.g., cataract removal most likely)

Grade 3	Full thickness corneal opacity	Two surgical procedures: CLET+ corneal transplant

Grade 4	Full thickness corneal opacity plus another noncorneal reason for visual loss (e.g., cataract)	Three surgical procedures: CLET+ corneal transplantation-corneal surgery (e.g., cataract removal)

Grade 0	Any grade of corneal opacity plus noncorneal irreversible visual loss (e.g., irreversible retinal pathology, advanced glaucoma)	No potential for gain: CLET performed to avoid globe removal

**Table 3:** Grading of ocular surface clinical characteristics.

	Ciliary hyperemia [50]	Central corneal epithelial opacity [50]	Central corneal epithelial irregularity [24]	Corneal epithelial integrity		Corneal superficial neovascularization [50]	
				Superficial punctate keratitis^*∗*^	Persistent epithelial defect area [24]	Area	Length
Grade 0	White conjunctiva	None	Normal/absent	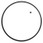	None	None	None

Grade 1	Widening of the vessels	Mild	Mild	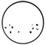	≤1/4	≤1/4	1 mm

Grade 2	Mild hyperemia	Moderate	Moderate	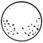	>1/4 and ≤1/2	>1/4 and ≤1/2	2-3 mm

Grade 3	Moderate hyperemia	Severe with faint pupil	Severe	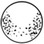	>1/2 and ≤3/4	>1/2 and ≤3/4	4-5 mm

Grade 4	Intense hyperemia	Severe with no visible pupil	N/A	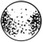	>3/4	>3/4	≥6 mm

Grade 5	N/A	N/A	N/A	>Grade 4	N/A	N/A	N/A

^*∗*^Modified from [51].
